# Preconceptional Resveratrol Supplementation Partially Counteracts Age-Related Reproductive Complications in C57BL/6J Female Mice

**DOI:** 10.3390/molecules26071934

**Published:** 2021-03-30

**Authors:** Marta Ziętek, Katarzyna Barłowska, Barbara Wijas, Ewa Szablisty, Atanas G. Atanasov, Jacek A. Modliński, Artur H. Świergiel, Silvestre Sampino

**Affiliations:** 1 Department of Experimental Embryology, Institute of Genetics and Animal Biotechnology of the Polish Academy of Sciences, ul. Postępu 36A, Jastrzębiec, 05-552 Magdalenka, Poland; m.zietek@igbzpan.pl (M.Z.); k.barlowska@igbzpan.pl (K.B.); b.wijas@igbzpan.pl (B.W.); e.szablisty@igbzpan.pl (E.S.); j.a.modlinski@igbzpan.pl (J.A.M.); 2 Department of Animal and Human Physiology, Faculty of Biology, University of Gdańsk, ul. Wita Stwosza 59, 80-308 Gdańsk, Poland; 3 Prof. Wacław Dąbrowski Institute of Agricultural and Food Biotechnology, ul. Rakowiecka 36, 02-532 Warsaw, Poland; 4 Ludwig Boltzmann Institute for Digital Health and Patient Safety, Medical University of Vienna, 1090 Vienna, Austria; atanas.atanasov@univie.ac.at; 5 Department of Biotechnology and Nutrigenomics, Institute of Genetics and Animal Biotechnology of the Polish Academy of Sciences, ul. Postepu 36A, Jastrzębiec, 05-552 Magdalenka, Poland; 6 Institute of Neurobiology, Bulgarian Academy of Sciences, Acad. G Bonchev Str. bl. 23, 1113 Sofia, Bulgaria; 7 Department of Pharmacognosy, University of Vienna, Althanstrasse 14, 1090 Vienna, Austria

**Keywords:** aging, natural compounds, resveratrol, reproduction, mice

## Abstract

Aging is associated with a drastic decline in fertility/fecundity and with an increased risk of pregnancy complications. Resveratrol (RES), a natural polyphenolic compound, has shown anti-oxidant and anti-inflammatory activities in both human and animal models, thus representing a potential therapeutic and prophylactic anti-aging supplement. Here, we investigated whether preconceptional resveratrol supplementation improved reproductive outcomes in mid-aged (8-month-old) and old (12-month-old) C57BL/6J female mice. Female siblings were cohoused and assigned to either RES or vehicle supplementation to drinking water for 10 consecutive weeks. Subsequently, females were mated with non-supplemented males and their pregnancy outcomes were monitored. RES improved mating success in old, but not in mid-aged females, and prevented the occurrence of delivery complications in the latter. These results indicate that preconceptional RES supplementation could partially improve age-related reproductive complications, but it was not sufficient to restore fecundity in female mice at a very advanced age.

## 1. Introduction

The current trend toward postponing childbearing is increasing in all developed countries in both men and women, who prefer to pursue social and career achievements rather than establish a family [1]. In women, aging is accompanied by a physiologic decline of fertility leading to menopause. Nowadays, improved welfare and assisted reproductive technologies allow women at advanced reproductive age to conceive children naturally or artificially [2,3]. Nevertheless, epidemiological and animal studies have shown that advanced maternal age (AMA) is associated with an increased risk of pregnancy complications, as well as with negative health outcomes in the offspring, including miscarriages and abortions, multisystem malformations, and neurodevelopmental disorders [4,5,6,7].

Recently, growing interest has been devoted to natural compounds as anti-aging dietetic supplements, including the flavonoid, resveratrol (RES) [8]. RES is a polyphenol produced by a vast variety of plants, including grapes, peanuts, and a variety of berries, among others [9]. A growing number of studies have indicated that RES has anti-inflammatory, anti-tumorigenic, antioxidant, and anti-aging properties, and improves general health in mammals [9,10,11,12,13,14]. Previous studies have evaluated the safety, pharmacokinetics, and metabolism of RES, and have reported it to be well tolerated in humans, even at high doses [15]. RES may protect against age-associated infertility in mice [16,17] and can decrease inflammatory responses in the placenta and embryonic oxidative stress [18], as well as improve fetal weight in compromised pregnancies [19], with no evidence of teratogenesis [20]. There is limited knowledge about the eventual beneficial or adverse effects of preconceptional RES supplementation on female age-related fertility and pregnancy complications. Therefore, the aim of this study was to test whether 10 weeks preconceptional RES supplementation may rescue reproductive decline in aged female mice. Offspring and pregnancy outcomes were evaluated in 8- and 12-month-old female siblings supplemented with either RES or vehicle (ethanol). The younger cohort were left to term delivery, whereas, in the 1-year-old cohort, pregnancies were interrupted at 12.5 days post coitum (dpc) to examine fetal and placental development. 

## 2. Results

**Figure 1:**
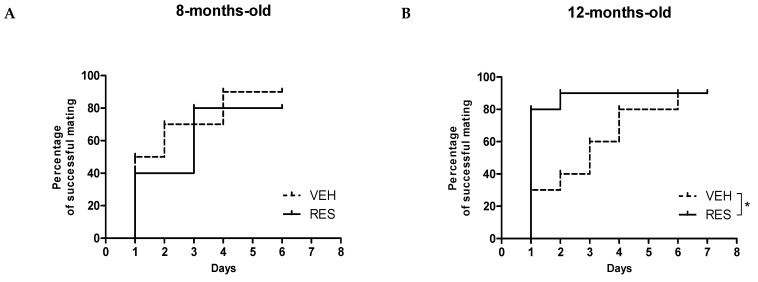
Percentage of females successfully mating (presence of the vaginal plug) over 7 days of daily encounters with males. Differences in time-to-mating between resveratrol (RES)- and vehicle (VEH)-supplemented females were analyzed with Gehan–Breslow–Wilcoxon survival test. No differences were observed in the 8-month-old cohort (**A**), whereas 12-month-old females (**B**) supplemented with RES mated faster than controls (* indicates *p* = 0.035). *n* = 10 females per experimental groups per age group.

First, we evaluated whether RES (or its vehicle ethanol) influenced the amount of liquid drunk by the mice. Although there was no effect of RES on liquid intake in the 8-month-old cohort, older females supplemented with RES drunk significantly more than their VEH-supplemented siblings at all timepoints analyzed (1st measurement, *p* = 0.02; 2nd measurement, *p* = 0.0009; 3rd measurement, *p* = 0.02; 4th measurement, *p* = 0.002; 5th measurement, *p* = 0.0006; with paired t-test). Treatment did not influence body weight during the supplementation period (F(3,35) = 1.89, *p* = 0.1498; with 2-way ANOVA). The cumulative percentage of females being successfully mated after 10 weeks of supplementation, thus presenting a vaginal plug after overnight male encountering, was similar between RES- and VEH-supplemented 8-month-old sibling females (Figure 1A).

**Figure 2:**
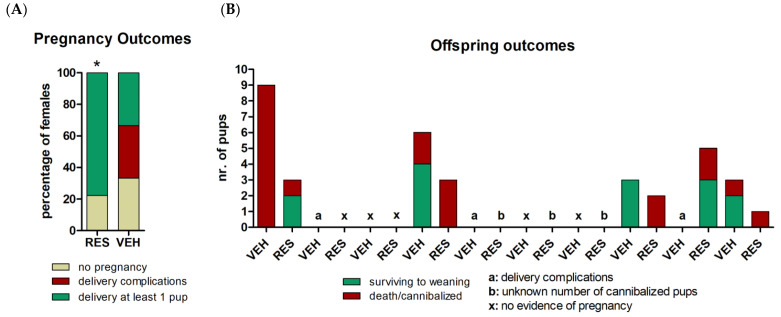
Pregnancy and offspring outcomes in 8-month-old RES- and VEH-supplemented female mice. (**A**) Percentage of pregnancies, delivery complications, and succsessful deliveries. Differences in delivery complications between RES- and VEH-supplemented females were analyzed with Chi square test (* indicates *p* = 0.329). (**B**) General view of the offspring outcomes observed in each female enrolled, including the number of pups surviving to weaning or death.

Conversely, 12-month-old females supplemented with RES mated significantly faster than age-matched controls (*p* = 0.035 with Gehan–Breslow–Wilcoxon test) (Figure 1B). As showed in Table 1, no significant differences were observed in fertility rate, neither at 0.5 dpc, nor at 18.5 dpc. In the youngest cohort, 50% of control females showed delivery complications and died during labor, whereas all pregnant RES-supplemented mothers delivered within 22 dpc, with no complications (*p* < 0.03 with Chi square test, Figure 2A). Considering only the females delivering at least one pup, litter size was decreased in 8-month-old females supplemented with RES, as compared with VEH-supplemented controls, although this difference was not statistically significant (RES, 2.8 ± 1.4; VEH, 5.2 ± 2.8; *p* = 0.342, with paired t-test).

No statistically significant effects of treatment were observed in cannibalization events, with 57% of RES-supplemented females cannibalizing at least one of their pups, while only 33.3% VEH control females displayed such behavior. Considering only the females which did not cannibalized any of their pups, there was no effect of treatment on pups’ survival to weaning, with a total of five pups obtained from RES-supplemented females, and nine pups from controls. Figure 2B depicts the delivery and offspring outcomes of each enrolled female.

In experiment 2, 12-month-old putative pregnant females were sacrificed 12.5 days after mating to collect conceptuses, however none of them were found in any of the RES- or VEH-supplemented females enrolled. 

## 3. Discussion

Considering the increasing trends toward delaying childbearing, there is a growing interest in establishing prophylactic and therapeutic strategies to counteract age-related reproductive failure. RES has been indicated as an anti-aging dietetic supplement with potential beneficial effects on age-related reproductive decline [21,22]. The present study shows that female mice supplemented with RES over 10 consecutive weeks displayed slightly better reproductive performances compared to not-supplemented sibling controls. On one hand, RES significantly reduced the occurrence of delivery complications in mid-aged females, while on the other it was not sufficient to counteract age-related reproductive failure in the older females’ cohort. Moreover, the effects of RES were also partial, in terms of the outcome considered, as there were not significant differences between groups concerning fertility and fecundity, whereas the occurrence of pregnancy complications was observed significantly more frequently in the control group compared to the RES-supplemented one. Moreover, RES was not sufficient to rescue fertility/fecundity in very old females under these experimental conditions. 

Advanced maternal age is reported to be associated with a range of maternal illness (such as hypertension and gestational diabetes), as well as with several pregnancy complications including fetal growth restriction, preeclampsia, placental defects, pre-term birth, and stillbirth [4,5,6,7]. Age-related failure in female reproductive ability is not exclusive to humans, and it is also evident in other mammalian species, such as the rat [23], the mouse [24], and large domestic species [25,26]. Previous studies indicated that C57 female mice display a reproductive decline starting at 6–8 months, which results in decreased pregnancy rate, increased fetal resorption and diminished litter size [27]. Such effects of ageing were also observed by us in an outbred strain [28] and in inbred C57 and BTBR T+ Itpr3tf/J mice [29]. In the present study, one-year-old females supplemented with RES were mated faster than controls. Considering that co-housed female mice tend to synchronize their oestrous cycles [30], this result indicates that the siblings supplemented with RES may have been more prone to mate compared to controls, or that RES may increase female attractiveness toward male breeders. Moreover, a beneficial effect of RES was observed concerning the occurrence of delivery complications in the younger cohort, which were not observed in RES-supplemented females, but occurred at a high rate in controls. Conversely, although 12-month-old RES-supplemented females had an increased chance of being mated, they were still not able to generate detectable offspring at 12.5 dpc. This suggests that a 10 weeks preconceptional supplementation may not be sufficient for RES to rescue all aspects of age-related reproductive decline, or that at an older age reproductive functions may be at a not-rescuable point. Overall, the present study retains some limitations related to the small sample size employed, combined with the low fertility of aged animals, which do not allow coming to consistent conclusions concerning the offspring outcomes. Nonetheless, the experiments were designed to control genetic and environmental variability among supplemented subjects, since pairs of sibling females were cohoused and assigned to either RES or VEH treatment. Therefore, although further animal and human studies will be needed, the present results suggest a beneficial effect of RES on age-related delivery complications.

## 4. Materials and Methods

### 4.1. Mice

C57BL/6J (C57) mice used in this study were purchased from Jackson Laboratory (Bar Harbor, ME, USA) and maintained at the Institute of Genetics and Animal Biotechnology PAS (Jastrzębiec, Poland) for the last 4 years, following a sibling mating breeding scheme. Mice were housed under a 12 h light-dark cycle, 19–23 °C, and 40–68% humidity, with standard pellet food and a source of water ad libitum. Sister females were co-housed in 26.5 × 20.7 × 14.0 cm^3^ cages, divided by a transparent and perforated PVC (polyvinyl) partition to overcome isolation-induced social stress. Bedding materials and environmental enrichment (including a nest) were provided to each subject on each side of the partition, together with food and one drinking bottle (two in total: one containing resveratrol (RES) and the other containing 0.9% ethanol (VEH), assigned to either sister respectively). This strategy allowed minimizing genetic and environmental confounding factors between the RES and VEH experimental groups. All experiments were conducted with the approval of the local institutional committee for animal welfare, and in line with Polish and European regulations.

### 4.2. Experimental Design

Two experiments were performed, each including 20 female mice (*n* = 10 females per treatment in each age cohort). In experiment 1, mice aged 22 weeks, were supplemented with RES or vehicle (VEH: ethanol) for 10 weeks, then mated and allowed to deliver spontaneously. In experiment 2, mice aged 38 weeks were supplemented in the same way, mated, and sacrificed 12.5 days after mating in order to collect conceptuses. During the first seven days of the experiments, mice were habituated to the new environment and to the 50 mL bottles. For the subsequent seven days, mice were habituated to 0.9% solution of ethanol (VEH) in drinking water, before the beginning of the supplementation period. The amount of liquid consumed by each mouse was measured every day to accommodate RES doses.

### 4.3. RES Supplementation to Drinking Water

RES (trans-Resveratrol; catalogue number 72862-72864, Stemcell Technologies Inc., Vancouver, Canada) was first dissolved in ethanol (50 mg/mL) and then diluted in drinking tap water (0.1 mg/mL RES – 0.9% *v/v* ethanol) and provided to mice based on a water intake measurement taken during the habituation periods, to obtain an average 16 mg/kg/day intake of RES. During the supplementation periods volumes of RES or VEH solutions consumed by each mouse were scored every 3–4 days, when mice received a fresh supply. Dosage of RES was selected based on previous studies [31].

### 4.4. Breeding

After 10 weeks of supplementation, the females were marked by ear cut and the cage separator was removed. Females were then introduced overnight to two 2–4-month-old stud males of the same strain, daily, for 6 consecutive days. The presence of a vaginal plug was checked each morning after overnight encountering and considered as day 0.5 dpc. Pregnancy at 18.5 dpc was scored by the presence of an evident belly swell. Gestational age >22 dpc and unsuccessful labor were considered as delivery complications.

### 4.5. Outcomes and Statistics 

Liquid intake was measured at five timepoints during the supplementation period, and differences between groups were analyzed by paired t-test. The effects of treatment and time on body weight before and after supplementation were analyzed by 2-way ANOVA. For assessment of time-to-mating success at 0.5 dpc, Gehan–Breslow–Wilcoxon survival tests was employed to compare experimental groups. Pregnancies at 0.5 and at 18.5 dpc are expressed as percentage of females and were analyzed by Chi square test. Delivery was classified as complicated when the female was not able to deliver the pups and died soon after. Delivery complications are expressed in percentage of females and analyses by Chi square test. Differences in litter size were analyzed by paired t-test between sister pairs. Differences in postnatal outcomes, such as pups’ survival and cannibalization, were expressed as percentage and analyzed by Fisher exact test. *P* < 0.05 was considered as statistically significant. All analyses were performed using GraphPad Prism version 5.0 (GraphPad Software Inc., San Diego, CA, USA) and R (available online: https://www.R-project.org/, accessed on 10 November 2020).

## 5. Conclusions

RES is a promising therapeutic/prophylactic compound in gerontology and reproductive medicine. The present study indicates that preconceptional RES supplementation could partially improve age-related reproductive complications, but it is not sufficient to rescue fecundity in female mice at a very advanced age. Long-term RES supplementation is known to improve ovarian function, increasing the ovarian follicular reserve and extending the ovarian life span in rats [32], as well as to improve the number of follicles in aged mice ovaries [33]. However, RES supplementation may also induce adverse effects on implantation and decidualization in both humans and animals [34]. Therefore, more research is needed to establish optimal doses and periods of RES supplementation to obtain a beneficial impact on reproduction, while preventing adverse effects on implantation and subsequent pregnancy.

## Figures and Tables

**Table 1:** Reproductive outcomes of 8-month-old C57BL/6J females supplemented with resveratrol and vehicle for 10 weeks. dpc: days post coitum.

	Evidence of Pregnancy at 0.5 dpc No. (%)	Evidence of Pregnancy at 18 dpc No. (%)	Litter Size at Delivery ^b^ *Mean ± SEM*	Offspring at 21 Days Postnatal No.
**Resveratrol**	9/10 (90%)	7/9 (78%)	2.8 ± 1.4	5
**Vehicle**	9/9 (100%) ^a^	6/9 (67%)	5.2 ± 2.8	9

^a^ One of the VEH-supplemented females died during the supplementation period. ^b^ Average litter size calculated considering females delivering at least one pup.

## Data Availability

The authors confirm that the data supporting the findings of this study are available within the article, and that raw data are available from the corresponding authors on request.

## Sample Availability

Samples of this work are available from the authors.
